# Enhanced protein-energy provision via the enteral route in critically ill patients: a single center feasibility trial of the PEP uP protocol

**DOI:** 10.1186/cc8991

**Published:** 2010-04-29

**Authors:** Daren K Heyland, Naomi E Cahill, Rupinder Dhaliwal, Miao Wang, Andrew G Day, Ahmed Alenzi, Fiona Aris, John Muscedere, John W Drover, Stephen A McClave

**Affiliations:** 1 Department of Medicine, Queen's University, 76 Stuart Street, Kingston, ON K7L 2V7, Canada; 2 Department of Community Health and Epidemiology, Queen's University, 76 Stuart Street, Kingston, ON K7L 2V7, Canada; 3 Clinical Evaluation Research Unit, Kingston General Hospital, 76 Stuart Street, Kingston, ON K7L 2V7, Canada; 4 Clinical Nutrition, Kingston General Hospital, Kingston, 76 Stuart Street, Kingston, ON K7L 2V7, Canada; 5 Department of Surgery, Queen's University, 76 Stuart Street, Kingston, ON K7L 2V7, Canada; 6 Department of Medicine, Louisville School of Medicine, 550 South Jackson Street, Louisville, KY 40202, USA

## Abstract

**Introduction:**

The purpose of this pilot study is to assess the feasibility, acceptability, and safety of a new feeding protocol designed to enhance the delivery of enteral nutrition (EN).

**Methods:**

In a prospective before and after study, we evaluated a new protocol compared to our standard feeding protocol. Innovative elements of the new protocol included setting daily volume based goals instead of hourly rate targets, initiating motility agents and protein supplements on Day 1, liberalizing the gastric residual volume threshold, and the option to use trophic feeds. Bedside nurses filled out questionnaires to assess the acceptability of the new approach and we assessed patients' nutritional and clinical outcomes.

**Results:**

We enrolled 20 mechanically ventilated patients who stayed in the Intensive Care Unit for more than three days in the before group and 30 such patients in the after group. On a scale where 1 = totally unacceptable and 10 = totally acceptable, 30 nurses rated the new protocol as 7.1 (range 1 to 10) and no incidents compromising patient safety were observed. In the before group, on average, patients received 58.8% of their energy and 61.2% of their protein requirements by EN compared to 67.9% and 73.6% in the after group (*P *= 0.33 and 0.13). When the subgroup of patients prescribed to receive full volume feeds in the after group were evaluated (n = 18), they received 83.2% and 89.4% of their energy and protein requirements by EN respectively (*P *= 0.02 for energy and 0.002 for protein compared to the before group). The rates of vomiting, regurgitation, aspiration, and pneumonia were similar between the two groups.

**Conclusions:**

This new feeding protocol seems to be safe and acceptable to critical care nurses. The adoption of this protocol may be associated with enhanced delivery of EN but further trials are warranted to evaluate its effect on nutritional and clinical endpoints.

**Trial registration:**

ClinicalTrials.gov NCT01102348

## Introduction

Several observational studies have described an association between inadequate feeding and poor clinical outcomes in critically ill patients [1-3]. In a multicenter observational study of 2,772 Intensive Care Unit (ICU) patients from 165 ICUs, Alberda and colleagues showed a significant inverse linear relationship between the odds of mortality and total daily calories received [4]. An increase of 1,000 calories per day was associated with reduced mortality (Odds Ratio for 60-day mortality 0.76 (95% Confidence Intervals (CI) 0.61 to 0.95, *P *= 0.014)) and an increased number of ventilator-free days (VFDs) (3.5 VFD, 95% CI, 1.2 to 5.9, *P *= 0.003). Since the main route of providing energy and calories was enteral nutrition, efforts to increase the enteral provision of macronutrients are warranted.

Repeated efforts over the past few years have not significantly improved the amount of calories delivered via the enteral route [5-7]. If we are to be successful at increasing the provision of calories and protein via the enteral route, a new paradigm is required. Historically, feeding protocols have been used to guide the delivery of enteral nutrition (EN) but they frequently utilize conservative, reactionary approaches to optimizing nutrition. For example, enteral feeds are started at low rates, are advanced slowly, and maintained at a target maintenance rate with no provisions to compensate for loss of feeding time due to frequent interruptions. Moreover, motility agents are only initiated after manifestations of delayed gastric emptying develop. The result is a form of iatrogenic malnutrition in which critically ill patients consistently receive less than their prescribed nutritional needs.

We propose a new approach that protocolizes an aggressive approach to providing EN and shifts the paradigm from reactionary to proactive followed by de-escalation if nutrition therapy is not needed. The key components of this new protocol are the following: 1) Starting feeds at the target rate based on increasing evidence that some patients tolerate starting nutrition at a higher rate of delivery and that slow start ups are not necessary [8,9]. For patients who are hemodynamically stable, we propose to shift from an hourly rate target goal to a 24-hour volume goal and give nurses guidance on how to make up this volume if there was an interruption for non-gastrointestinal reasons [10]. This *volume-based *goal represents a significant shift in practice from traditional hourly rate goals in which nurses can increase the hourly rate depending on how many hours they have left in the day to ensure that the patient receives the 24-hour volume within the day. 2) For patients who are deemed unsuitable for high volume intragastric feeds, we provide an option to initiate *trophic feeds *at a low volume of a concentrated feeding solution. By *trophic*, we mean a minimal volume of EN designed to maintain gastrointestinal structure and function, not designed to meet the patients caloric or protein needs. When deemed suitable, trophic feeds can be advanced to full feeds. 3) Rather than wait for a protein debt to accumulate because of inadequate delivery of EN, protein supplements are prescribed at initiation of EN and can be discontinued if EN is well tolerated. 4) We propose to start motility agents at the same time EN is started with a re-evaluation in the days following to see if it is necessary and we raised our gastric residual volume threshold from 200 to 250 ml. It has been shown in one randomized trial that a feeding protocol that starts a motility agent empirically at the time of initiation of feeds and uses a higher threshold for a critical gastric residual volume (250 ml) improves nutritional adequacy [11].

Since the bedside nurses initiate and utilize feeding protocols to achieve target goals, we will couple this newer generational feeding protocol with a comprehensive nurse-directed nutritional educational intervention that will focus on its safe and effective implementation. This focus on nursing nutrition education represents a major shift away from traditional education which has focused on dietitians and physicians.

We hypothesize that the combination of these components will safely improve the provision of energy and protein compared to usual care. We postulate that this increased provision of calories and protein may translate into improved clinical outcomes, particularly for the patients at the extremes of weight, but the current study is not powered to demonstrate such a difference. The purpose of this pilot study is to assess the feasibility, acceptability, and safety of this new feeding protocol, "*The Enhanced Protein-Energy Provision via the Enteral Route in Critically Ill Patients: The PEP uP Protocol."*

## Materials and methods

This is a prospective before and after study conducted in a 21-bed medical surgical tertiary care ICU with a usual nurse-to-mechanically-ventilated-patient ratio of one-to-one that admits approximately 1,000 patients per year. All care in the ICU is directed by an attending intensivist. The clinical staff in the ICU also includes a full time registered dietitian. Enteral feeds are guided by a standard feeding protocol specified by pre-printed ICU admission orders. The admitting physician has the option of initiating the enteral feeding protocol or keeping the patient nil per os (NPO). If EN is ordered, the bedside nurse initiates a standard polymeric feeding formula at 25 ml/hr once the position of the feeding tube is confirmed. Gastric residual volumes are monitored every four hours and in the absence of a gastric residual volume above 200 ml, feeds are advanced to their target hourly rate by 25 ml/hr every four hours. In the setting of persistent (two or more consecutive) high gastric residual volumes, feeds are reduced by 25 ml/hr and the nurse is instructed to ask the physician to order gastrointestinal prokinetic agents (metoclopramide first, erythromycin as the second line therapy), if appropriate. The dietitian usually assesses the nutritional needs and prescribes the appropriate solution and hourly target rate within 24 to 48 hours of admission. In the summer of 2008, we evaluated the efficacy of our standard protocol in a cohort of eligible patients.

Starting in January 2009, we implemented our new protocol combined with our nursing educational intervention. The concepts and the details of the PEP uP Protocol were presented at our multidisciplinary critical care rounds and augmented by small group educational in-servicing for bedside nurses. We operationalized the protocol with a pre-printed order form and a series of bedside algorithms and instructions. On the pre-printed order form, there were tick boxes for the admitting physician to choose *full volume feeds*, *trophic feeds*, or *NPO*. Beside each tick box, there were indications and contraindications listed for each selection. If an admitting physician selected *NPO*, they had to fill in a reason or justification. A sample of justifications was provided on the form. To simplify the administration of this protocol, we chose one feeding formula to be used initially. We chose a semi-elemental, concentrated feeding solution that would be useful in both full volume and trophic fed patients (Peptamen 1.5^®^, Nestle, North York, Ontario, Canada). The dietitian could suggest changes to this formula after the protocol was started based on further assessment. There was also a tick box to initiate a motility agent in the absence of contra-indications (Metoclopramide, 10 mg intravenously q 6 h, with dosage adjustment in renal failure) and to initiate a protein supplement (Beneprotein^®^, Nestle Healthcare, Minneapolis, MN, USA, 14 gms bid providing 24 gm protein per day). Nurses were given instructions on how to set the hourly rate based on the 24-hour volume prescribed. For example, if the total goal for the day was 1,500 ml of a nutritional solution to meet their caloric requirement, then the hourly rate would be 62.5 ml/hr. If feeds were held for several hours while the patient underwent a radiological procedure and now there are nine hours left in the day and the patient has only received 400 ml, the new rate would be (1,100 ml/9 hrs) 122 ml/hr for the remaining 9 hrs. Beginning the next day, the target would shift back to 62.5 ml/hr. We arbitrarily set a limit of a maximum of 150 ml/hr for PEP uP Protocol patients. We increased the threshold gastric residual volume to 250 ml from 200 ml and since patients were already on metoclopramide as per the protocol, erythromycin was added if the threshold gastric residual volume of 250 ml persisted.

We encouraged nurses to calculate the nutritional adequacy (% received over % calories prescribed) daily and report it during daily multidisciplinary rounds. Given the concerns raised about the possibility of precipitating a *refeeding syndrome *with the aggressive feeding strategy, the protocol also required frequent assessment of electrolytes, magnesium, phosphorus, and calcium during the first 72 hours of admission of all patients.

These protocol documents (that is, pre-printed order, algorithm for advancing feed, and algorithm for calculating rate of administering feed as per 24-hour volume) and a slide presentation coupled with educational reminders (posters and bedside notices) and practice helps (tool to remind nurse to measure and report nutritional adequacy) were made available to all nurses working in our unit, in bedside manuals and on the local intranet. Beginning 2 February 2009, we formally introduced the protocol and evaluated its adoption in the next series of eligible patients. Nurses attending to patients enrolled in the after phase of the study received daily bedside academic detailing (one on one instruction) by the ICU Dietitian.

Our study patient population consisted of consecutive mechanically ventilated adult patients (>18 years old) who remained in ICU for more than 72 hours. In the before group, we enrolled 20 patients prospectively. To ensure a larger number available for evaluation of the new protocol, we enrolled 30 patients in the after group.

In both groups, we retrospectively collected the following information related to the prospectively enrolled patients from their hospital chart: admission category (surgery vs. medical), primary admission diagnosis, sex, age, weight, height, and Acute Physiology And Chronic Heath Evaluation II (APACHE II) score [12]. We recorded daily the goal calories and protein as per the dietitian's assessment, the type and amount of nutrition received, and morning blood glucose levels for a maximum of 12 days or until death or discharge from ICU. We followed patients while in hospital for a maximum of 60 days and reported on their ICU and hospital outcomes at 60 days. Two physicians independently reviewed the charts of all enrolled patients to determine whether episodes of vomiting, regurgitation, macroaspiration (visually noted gastric secretions when suctioning the endotracheal tube), and ventilator-associated pneumonia had occurred. Pneumonia was defined using a definition and process that has been described elsewhere [13].

The primary outcome of this pilot study was the feasibility of the new feeding protocol as judged by a nursing questionnaire that evaluates their opinion of its safety and acceptability. We asked about the acceptability of each of the novel parts of the feeding protocol and the overall protocol using a scale where 1 = totally unacceptable and 10 = totally acceptable. This evaluation questionnaire was administered to the bedside nurse involved in caring for a patient on the new protocol (in the after group). Secondary outcomes included nutritional endpoints (adequacy of EN and timeliness of initiation of EN) and safety endpoints (episodes of vomiting, aspiration and pneumonia). In addition, one of the investigators reviewed the charts to determine if there were any undetected serious adverse events related to the nutritional management of the patient and whether the initial prescription of full volume, trophic feeds, or NPO was appropriate.

No formal sample size calculation was done as the primary purpose of this before and after study was to evaluate the feasibility and safety of the new protocol and not its effect on mortality or length of stay. Categorical variables are reported as counts and percents and compared between cohorts by the Fisher's Exact test. Length of stay variables are described by medians and quartiles and compared by the log-rank test. Other continuous variables are described by their means and standard deviations and compared by the Wilcoxon-Mann-Whitney test To assess nutritional adequacy, the total amount of energy or protein received from either EN or parenteral nutrition (PN), inclusive of propofol, was divided by the amount prescribed as per the baseline assessment and expressed as a percentage. For the purposes of this evaluation of this enteral feeding protocol, we compared adequacy from EN sources between the two groups as the primary outcome over the first seven ICU days. Patients receiving no EN were excluded from this analysis of EN adequacy. We also report a subgroup analysis of the EN adequacy of just those patients indicated to receive full volume feeds, as per the order on the admission pre printed order forms. Statistical analysis was completed using SAS v9.1.3 (SAS Institute Inc., Cary, NC, USA). All tests were two-sided with statistical significance considered as a *P*-value < 0.05. Institutional ethics approval was obtained from the Health Sciences Research Ethics Board at Queen's University, Kingston, Ontario, Canada. The need for informed patient consent was waived given the nature of this study (a system-level quality improvement study).

## Results

In the before group, we screened consecutive admissions and enrolled 20 mechanically ventilated patients who stayed in the ICU more than three days. In the after group, we again screened consecutive patients but this time enrolled 30 patients who remained in the ICU more than three days. The characteristics and outcomes of all enrolled patients are shown in Table 1. There were no significant differences in baseline characteristics between study groups, but survivors in the after group tended to have a shorter ICU and hospital length of stay than survivors in the before group.

**Table 1:** Patient characteristics and clinical outcomes

	Before (n = 20)	After (n = 30)	*P*-value*
**Age (years)**			0.39
Mean ± SD	59.5 ± 17.3	64.4 ± 16.7	
**Gender**			1.0
Female	9 (45.0%)	13 (43.3%)	
Male	11 (55.0%)	17 (56.7%)	
**Admission category**			0.86
Medical	16 (80.0%)	22 (73.3%)	
Surgical: Elective	3 (15.0%)	7 (23.3%)	
Surgical: Emergency	1 (5.0%)	1 (3.3%)	
**Admission diagnosis**			0.07
Cardiovascular/vascular	7 (35.0%)	7 (23.3%)	
Respiratory	3 (15.0%)	8 (26.7%)	
Gastrointestinal	0	5 (16.7%)	
Neurologic	1 (5.0%)	2 (6.7%)	
Sepsis	5 (25.0%)	1 (3.3%)	
Trauma	3 (15.0%)	1 (3.3%)	
Metabolic	0	2 (6.7%)	
Renal	0	2 (6.7%)	
Other	1 (5.0%)	2 (6.7%)	
			
**APACHE II score**			0.73
Mean ± SD	20.7 ± 6.9	21.3 ± 7.3	
**Presence of ARDS**			1.0
No	20 (100.0%)	29 (96.7%)	
Yes	0	1 (3.3%)	
**Length of ICU stay (days) **†			0.14
Median(IQR)	17.9 (11.2 to 47.8)	8.5 (4.9 to 12.7)	
**Length of hospital stay (days) **†			0.02
Median (IQR)	57.4 (36.3 to Und.‡)	22.9 (18.0 to 46.6)	
**Length of mechanical ventilation (days) **†			0.06
Median(IQR)	11.8 (7.6 to 43.3)	5.8 (3.6 to 8.6)	
**Patient died within 60 days of ICU admission**			0.16
No	18 (90.0%)	21 (70.0%)	
Yes	2 (10.0%)	9 (30.0%)	

* Age and Apache II score were tested by the Wilcoxon-Mann-Whitney test, categorical variables were tested by Fisher's exact test, and length of stay variables were tested by the log-rank test.

† Based on 60-day survivors only. Time before ICU admission is not counted.

‡ Undefined because 44% (8/18) of surviving patients remained in hospital 60 days after ICU admission.

SD, standard deviation; IQR, interquartile range; APACHE, acute physiology and chronic health evaluation score; ARDS, acute respiratory distress syndrome; ICU, intensive care unit.

On average, across the two time periods, study patients were prescribed 24 to 26 kcal/kg and 1.1 to 1.2 grams/kg of protein by the ICU dietitian. Details of the nutritional assessment and the nutritional prescription of study patients can be seen in Table 2. In the before group, 12 patients were prescribed initial enteral feeds while eight patients were kept NPO. In the after group, 18 patients were initially prescribed full volume feeds, six were prescribed trophic feeds, and six were kept NPO. In the after group, the reasons patients were kept NPO included impending operation, post gastric surgery and no feeding tube in place, post-op bowel ischemia, impending extubation, high nasogastric drainage, and no reason provided. Based on a chart review, 2/6 (33%) of these patients were kept NPO without adequate justification. With one exception, all patients in the after group eventually received EN (eight (100%) NPO patients in the before group and five of six (83.3%) NPO patients in the after group were fed EN).

**Table 2:** Patient nutrition assessment information

	Before (n = 20)	After (n = 30)	*P*-value
**Height**			0.34
Mean ± SD	1.7 ± 0.1	1.7 ± 0.1	
**Weight**			0.92
Mean ± SD	79.3 ± 26.8	81.8 ± 27.3	
**Body Mass Index (kg|m2)**			0.75
Mean ± SD	26.9 ± 6.7	29.5 ± 11.2	
**Weight used in calculation of nutrition prescription**			0.001
Actual Body Weight (ABW)	10 (50.0%)	21 (70.0%)	
Estimated	7 (35.0%)	5(16.7%)	
Adjusted by 25% (ABW × 0.25 + Ideal body weight)	1 (5.0%)	4 (13.3%)	
No assessment completed	1 (5.0%)	0	
Other, please specify	1 (5.0%)	0	
**Method used to calculate energy requirement**			0.29
Ireton-Jones Equation	17 (85.0%)	29 (96.7%)	
Weight based: 30-35 Kcal/Kg	1 (5.0%)	1 (3.3%)	
Other, please specify	2 (10.0%)	0	
**Prescribed energy intake (kcals)**			0.19
Mean ± SD	1983.8 ± 504.1	1831.2 ± 293.3	
**Prescribed protein intake (g)**			0.29
Mean ± SD	88.6 ± 20.2	83.2 ± 13.4	
**Prescribed energy intake by weight (kcals|kg)**			0.18
Mean ± SD	26.4 ± 6.6	24.0 ± 5.9	
**Prescribed protein intake by weight (g|kg)**			0.13
Mean ± SD	1.2 ± 0.4	1.1 ± 0.2	

Variable pertinent to the nutritional assessment of study patients. SD, standard deviation; ABW, actual body weight; IBW, Ideal body weight

The average time to starting EN was 16.0 hours in the before group and 17.7 hours in the after group (*P *= 0.72) (see Table 3). The majority of study patients were started on enteral feeds within the first 24 hours in both groups (16/20 (80%) before and 23/30 (82.1%) after). The average daily amount of gastric residual volume is 243 (range 0 to 1,740) in the before group, and 231 (range 0 to 1,698) in the after group (*P *= 0.22). In the before group, no patients received motility agents or protein supplements on Day 1 of admission whereas in the after group, the number of patients that on Day 1 of admission received protein supplements and motility agents was nine (30.0%) and seven (23.3%) respectively (see Table 3). The average duration of use of the protein supplements was four (range: 1 to 12) days and of the motility agents was 5.9 (range: 2 to 12) days. In the after group, more patients overall tended to receive motility agents (80.0% vs. 55.0%, *P *= 0.11) and protein supplements (63.3% vs. 35.0%, *P *= 0.08) compared to the before group (see Table 3).

**Table 3:** Nutritional outcomes

	Before (n = 20)	After (n = 30)	*P*-value
**Adequacy of calories from total nutrition **†‡			0.23
Mean ± SD	71.7% ± 32.5%	80.3% ± 27.0%	
**Adequacy of protein from total nutrition **†‡			0.13
Mean ± SD	61.2% ± 29.3%	73.6% ± 28.6%	
**Adequacy of calories from EN **‡			0.23
Mean ± SD	58.8% ± 31.4%	70.2% ± 26.3%	
**Adequacy of protein from EN **‡			0.08
Mean ± SD	61.2% ± 29.3%	76.1% ± 25.5%	
**Type of nutrition**			0.16
EN only	18 (90.0%)	29 (96.7%)	
PN only	0	1 (3.3%)	
EN + PN	2 (10.0%)	0	
**Initiation of EN**			0.96
Prior to ICU admission	1 (5.0%)	2 (7.1%)	
0 to 24	15 (75.0%)	21 (75.0%)	
>24 to 48	4 (20.0%)	3 (10.7%)	
>48 to 72	0	1 (3.6%)	
>72	0	1 (3.6%)	
**Time of initiation of EN (hours)**			0.72
Mean ± SD	16.0 ± 13.8	17.7 ± 16.9	
**Use of motility agents (patient-day level)**			0.70
n/N (%)	78/192 (40.6%)	104/243 (42.8%)	
**Use of motility agents (patient level)**			0.11
n/N (%)	11/20 (55.0%)	24/30 (80%)	
**Started motility agents on the first ICU day¶**			0.03
n/N (%)	0	7/30 (23.3%)	
**Use of supplemental protein**			0.08
n/N (%)	7/20 (35.0%)	19/30 (63.3%)	
**Started supplemental protein on the first ICU day£**			0.007
n/N (%)	0	9/30 (30.0%)	
**Morning blood glucose (mmol/l)**			1.0
mean ± SD	8.0 ± 0.9	8.1 ± 1.0	
**%Patient-days with blood glucose>10 mmol/l**			0.60
mean ± SD	16.4% ± 10.1%	16.2% ± 12.8%	

† includes propofol, EN, and PN when there is a contraindication for EN

‡ includes the first seven ICU days only

¶ The average duration of use of motility agents is 5.9 days in the after group.

£ The average duration of use of supplemental protein is four days in the after group.

SD, standard deviation; EN, enteral nutrition; PN, parenteral nutrition; ICU, intensive care unit

**Figure 1:**
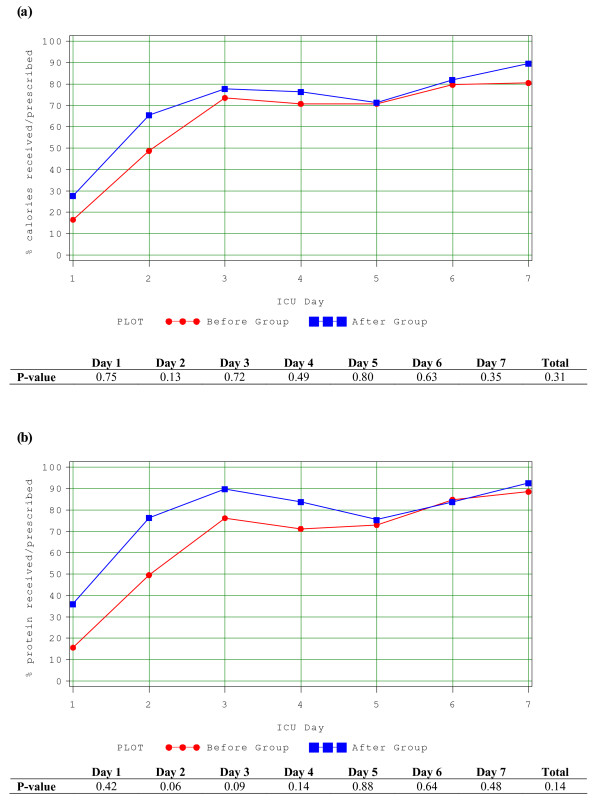
**Adequacy of calories and proteins from EN**. **(a) **Amount of calories received over the amount prescribed during the first seven days between the two study groups. **(b) **Amount of protein received over the amount prescribed during the first seven days between the two study groups.

**Figure 2:**
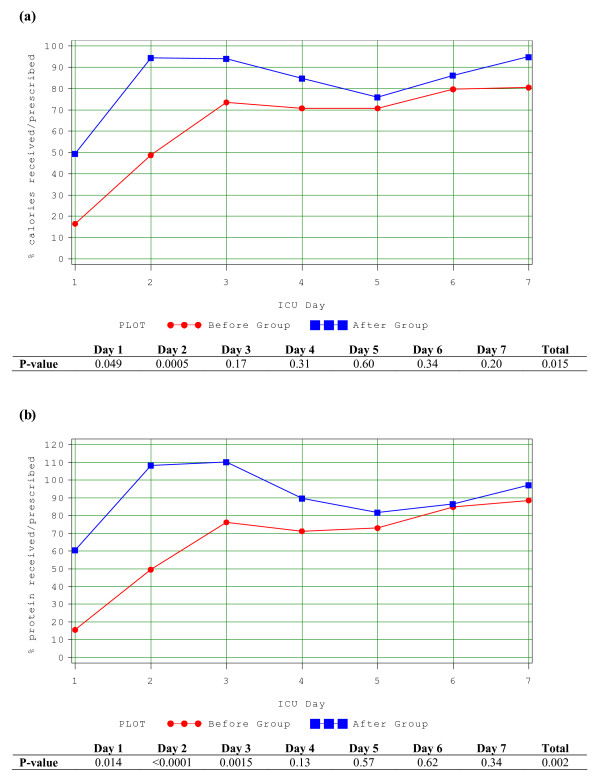
**Adequacy of calories and proteins from EN (before group vs. after group on full volume feeds)**. **(a) **Amount of calories received over the amount prescribed during the first seven days in patients in before group compared to those on full volume feeds in the after group. **(b) **Amount of protein received over the amount prescribed during the first seven days in patients in before group compared to those on full volume feeds in the after group.

Of the 29 patients who received EN in the after group, only 22 (76%) started on Peptamen 1.5. The average duration of use of Peptamen 1.5 was 3.0 days, with standard deviation of 1.8 days. Only one patient in the before group was started on Peptamen 1.5, the majority of the remaining were started on a polymeric solution. In the before group, on average, patients received 58.8% (range 0 to 116%) of their energy and 61.2% (range 0 to 104%) of their protein requirements from EN. In the after group, patients received 67.9% (range 0 to 139%) and 73.6% (range 0 to 119%) of their energy and protein requirements respectively (*P *= 0.33 and 0.13) (See Figure 1a, b) from EN. When only the subgroup of patients prescribed to receive full volume feeds were evaluated (n = 18), they received 83.2% (range 47.9% to 139%) and 89.4% (range 43.2% to 119%) of their energy and protein requirements respectively (See Figure 2a, b). Intake was statistically significantly increased in the early part of the ICU stay in the after group (Days 1 and 2 for calories and Days 1, 2 and 3 for protein). By Day 2, on average, patients in the after group indicated to receive full volume feeds had received >90% of their protein and energy requirements. No patient in the before group received parenteral nutrition; one patient in the after group received it for two days.

There was no increase in complications related to the PEP uP Protocol. The rates of vomiting, regurgitation, aspiration, and ventilator-associated pneumonia (VAP) were similar between the two groups (see Table 4). In the before group, 13 (65%) patients ever had diarrhea while 17 (57%) patients in the after group ever had diarrhea during their first 12 ICU days (*P *= 0.78). There were no documented electrolyte complications in the after group. Morning blood glucose (8.0 ± 0.9 vs. 8.1 ± 1.0 mmol/L) and the proportion of measurements spent above 10 mmol/L (16.4% vs 16.2%, *P *= 0.60) were similar between the two groups.

**Table 4:** Complications

	Before (n = 20)	After (n = 30)	*P*-value
**Vomiting**			0.38
No	17 (85%)	28 (93.3%)	
Yes	3 (15%)	2 (6.7%)	
**Regurgitation**			0.16
No	18 (90%)	30 (100%)	
Yes	2 (10%)	0	
**Macro aspiration**			0.16
No	18 (90%)	30 (100%)	
Yes	2 (10%)	0	
**Pneumonia (48 hours after ICU admission)**			0.45
No	15 (75%)	26 (86.7%)	
Yes	5 (25%)	4 (13.3%)	

ICU, intensive care unit

Thirty nurses filled out the evaluation form on the safety and acceptability of the protocol. Four of the 30 (13%) indicated that they witnessed an event or incident that, in their opinion, compromised patient safety. These incidents were recorded in open text as the nurse perceptions that the patient was getting too much volume and concerns about new feeding pumps that had nothing to do with the protocol but unfortunately were deployed the same time we evaluated the new protocol. There were no indications from the nurses that patients had regurgitated or aspirated while on this feeding protocol. Nurses rated the acceptability of the protocol and its individual components as follows: the acceptability of the 24-hour volume based target was 7.0 (range 1 to 10); the acceptability of starting at a high hourly rate was 5.9 (range 1 to 10); the acceptability of starting motility agents right away was 7.4 (1 to 10); the acceptability of starting protein supplements right away 7.6 (1 to 10); and the acceptability of the overall protocol was 7.1 (range 1 to 10) on a scale where 1 = totally unacceptable and 10 = totally acceptable.

## Discussion

Worldwide, there is considerable controversy around the optimal amount of and route of feeding in critically ill patients. However, our recent observational research shows that the amount of energy and protein received during the early stages of ICU admission impacts on patient mortality [4]. A review of current practice in ICU patients worldwide shows that the actual amounts of energy and protein delivered by standard hospital nutrition protocols is well below what is prescribed [14]. Some will use these data to argue for the early utilization of parenteral nutrition to make up for the caloric/protein debt due to inadequate EN [15]. However, the evidence suggests, from several sources, that enteral nutrition is superior to PN [16-18]. If we are going to establish EN as the cornerstone of nutrition therapy in ICU, we need to drastically change our approach to providing EN.

What this paper introduces is a new philosophy for delivery of EN, where the clinician starts at near maximal therapy, optimizing as many strategies as possible. This means the clinician starts at goal rate with automatic initiation of prokinetic agents, uses a small peptide formula that would be tolerated by the most number of patients, optimizes protein delivery with early supplementation, minimizes interruptions by setting a higher gastric residual volume threshold, and promotes volume-based feeding so that nurses can make up for lost time due to diagnostic tests and surgical/endoscopic procedures. The goal is to optimize chances for tolerance, prevent ileus, shorten the time frame to achieve goal feeding, and take advantage of the window of opportunity early after admission to the ICU to change outcome with the EN. This philosophy is a radical departure from conventional provision of nutrition where clinicians typically start at a low rate of infusion, ramping up slowly, cautiously evaluating tolerance and adding prokinetic agents only after evidence of ileus is already present.

Although the current study is small and utilizes a weak study design, its value is in the new ideas it espouses to maximize EN delivery to critically ill patients. Based on nurse-rated questionnaires and a retrospective chart review, we conclude that the new PEP uP Protocol is safe, feasible, and acceptable to nurses working in our ICU. Moreover, for those patients who were prescribed volume based feeds, they achieved almost 90% of their prescribed protein and energy requirements. These gains were achieved with volume-based feeds despite the fact that they started EN the same time as the before group. Thus it appears that this protocol may enhance the delivery of enteral nutrition; however, this finding requires testing in a prospective randomized fashion. We acknowledge that there is evidence that aggressive, intragastric EN can cause harm [19]. Hence, we monitored our patients carefully for the negative consequences of EN and no increase in complications was observed. In fact, we observed a statistically significant reduction in hospital length of stay with this new protocol. However, it was not the purpose of this study to compared clinical outcomes. Our sample size was too small to properly evaluate clinically important outcomes and larger studies of a higher methodological quality are required to evaluate this protocol before clinical recommendations can be made. Furthermore, the generalizability of this study protocol to surgical critically ill patients, especially those with GI surgery, is limited inasmuch as less than 25% of patients had a surgical admission diagnosis. Finally, we operationalized this new protocol in an ICU with 1:1 nurse patient ratio. In ICUs with less intensive nursing resources, they may have even greater challenges utilizing this protocol successfully.

In implementing this protocol at our site, we observed several challenges which decreased the effectiveness of the protocol. First, although motility agents and protein supplements were meant to start on Day 1 on all PEP uP Protocol patients, in reality, this only happened in about one-third of patients. Also, while we intended for a ready to use, easily absorbed semi-elemental diet to be prescribed at initiation of the feeding protocol, this did not happen in 25% of the patients. Finally, despite our introduction of the concept of trophic feeds, as an alternative to *NPO*, 20% of protocolized patients were still ordered *NPO *at the outset of their ICU admission, some without adequate justification. These deficiencies highlight the need for improved training and education on these points to ensure better compliance with these new ideas. To the extent that we are more successful at their implementation, we will have greater success with EN adequacy than currently reported. Furthermore, we can increase the maximal hourly rate of the 24-hour volume based feeds (from 150 to 200 ml/hr) and/or increase the threshold gastric residual volume from 250 to a higher amount and these changes will likely also increase EN delivery.

We believe that the best way forward to implement these novel changes to the way we deliver EN is in the context of a feeding protocol. Such nurse-driven bedside feeding protocols have been shown to be effective in reducing interruptions and improving nutritional adequacy [20]. However, critical care nurses working in an intensive care unit that utilize feeding protocols describe a low level of autonomy, decision making, and knowledge related to enteral feeding practice [21]. Nurses reported that they felt that the guideline was to be followed and consequently there was a low level of practice-based inquiry because 'you just follow the guideline' [22]. Following the guideline resulted in reasonably consistent practice but a low level of initiative for proactive decision making and interdisciplinary collaboration regarding nutrition support practices. We posit that our nurses may be relatively disengaged from the process of EN delivery and this may represent a barrier to successful feeding with our current feeding protocols. The current PEP uP Protocol, coupled with a nurse-directed educational intervention may overcome this barrier by giving the bedside nurse more information related to early enteral feeding (in the slide presentation and bedside reminders) and more latitude in calculating the required amounts (24-hour volume feeds concept).

## Conclusions

Whereas significant underfeeding of critically ill patients occurs around the world, this new feeding protocol has the potential to dramatically improve nutrition practice world wide. This current study is valuable in that it introduces some key philosophical and practical changes to how enteral nutrition is prescribed and delivered to critically ill patients. We have shown, in the context of a single-center before and after study, that this new protocol, the PEP uP Protocol, is safe, feasible, and may improve nutritional adequacy. This important single center study justifies the need for a larger multicenter, multinational RCT to further determine the safety and efficacy of this novel approach to feeding the critically ill patient.

## Key messages

• Traditional enteral feeding protocols do not adequately provide sufficient protein and calories to critically ill patients.

• A new feeding protocol that challenges traditional assumption and incorporates innovative ideas on maximizing the benefits and minimizing the risks of EN is needed.

• The PEP uP Protocol uses 24-hour volume-based feeding goals instead of hourly rate targets, initiating motility agents and protein supplements on Day 1, liberalizing the gastric residual volume threshold, and the option to use trophic feeds.

• In this before and after pilot study, we demonstrate that this new feeding protocol seems to be safe and acceptable to critical care nurses.

• The adoption of this protocol may be associated with enhanced delivery of EN but further trials are warranted to evaluate its effect on nutritional and clinical endpoints.

## Abbreviations

APACHE II: acute physiology and chronic health evaluation score; CI: confidence interval; EN: enteral nutrition; ICU: Intensive Care Unit; NPO: nil per os; PN: parenteral nutrition; VAP: ventilator-associated pneumonia; VFD: ventilator-free days.

## Competing interests

Daren K Heyland has received research grants and a speaking honorarium from Nestle. All other authors have no competing interests to declare.

## Authors' contributions

DKH was responsible for the initial design of the study and writing of the manuscript. JM, AA and FA were responsible for data collection. MW and AGD conducted the analysis. JM, JWD, SAMC, RD and NEC provided input on the design and interpretation of the study. All authors have contributed to, seen, and approved this manuscript.
